# Linking Cell Dynamics With Gene Coexpression Networks to Characterize Key Events in Chronic Virus Infections

**DOI:** 10.3389/fimmu.2019.01002

**Published:** 2019-05-03

**Authors:** Mireia Pedragosa, Graciela Riera, Valentina Casella, Anna Esteve-Codina, Yael Steuerman, Celina Seth, Gennady Bocharov, Simon Heath, Irit Gat-Viks, Jordi Argilaguet, Andreas Meyerhans

**Affiliations:** ^1^Infection Biology Laboratory, Department of Experimental and Health Sciences (DCEXS), Universitat Pompeu Fabra, Barcelona, Spain; ^2^CNAG-CRG, Center for Genomic Regulation (CRG), Barcelona Institute of Science and Technology, Barcelona, Spain; ^3^Universitat Pompeu Fabra, Barcelona, Spain; ^4^Cell Research and Immunology Department, Tel Aviv University, Tel Aviv, Israel; ^5^Marchuk Institute of Numerical Mathematics, Russian Academy of Sciences, Moscow, Russia; ^6^Institute for Personalized Medicine, Sechenov First Moscow State Medical University, Moscow, Russia; ^7^Institució Catalana de Recerca i Estudis Avançats (ICREA), Barcelona, Spain

**Keywords:** systems biology, cell dynamics, coexpression networks, WGCNA, DCQ, LCMV, chronic infection

## Abstract

The host immune response against infection requires the coordinated action of many diverse cell subsets that dynamically adapt to a pathogen threat. Due to the complexity of such a response, most immunological studies have focused on a few genes, proteins, or cell types. With the development of “omic”-technologies and computational analysis methods, attempts to analyze and understand complex system dynamics are now feasible. However, the decomposition of transcriptomic data sets generated from complete organs remains a major challenge. Here, we combined Weighted Gene Coexpression Network Analysis (WGCNA) and Digital Cell Quantifier (DCQ) to analyze time-resolved mouse splenic transcriptomes in acute and chronic Lymphocytic Choriomeningitis Virus (LCMV) infections. This enabled us to generate hypotheses about complex immune functioning after a virus-induced perturbation. This strategy was validated by successfully predicting several known immune phenomena, such as effector cytotoxic T lymphocyte (CTL) expansion and exhaustion. Furthermore, we predicted and subsequently verified experimentally macrophage-CD8 T cell cooperativity and the participation of virus-specific CD8^+^ T cells with an early effector transcriptome profile in the host adaptation to chronic infection. Thus, the linking of gene expression changes with immune cell kinetics provides novel insights into the complex immune processes within infected tissues.

## Introduction

A virus infection of a host organism represents a major perturbation from homeostasis. It is temporary limited in case of an acute infection or maintained in a chronic infection. Nonetheless, in both types of virus infections, a large number of the host genes of a lymphatic tissue in which the immune response is initiated may be differentially expressed compared to the healthy steady state indicating the enormous complexity of the overall host protection response (1, 2). To define the key processes that determine virus infection fates, and to understand the underlying mechanisms, most analyses have concentrated on few immune cell subtypes or regulatory factors, without addressing the interactions between them [reviewed in (3)]. Higher resolution techniques like mass cytometry, single-cell technologies and mass spectrometry were then used to further characterize cell subtype populations (4–6) or fine-map intracellular processes of selected cell types (7, 8). The respective data demonstrated a large degree of functional diversity even within virus-specific immune cell subtypes and characterized specific functional cell states (9, 10).

An alternative, holistic strategy to analyse virus-induced host perturbations is to apply a high resolution technique like RNA-Seq for capturing all processes within a complete organ. This strategy however has the disadvantage that the use of total organ RNA eliminates all information about organ cell type composition at the time of analysis and cell origin of the RNAs. Nonetheless, recent work using infection of mice with influenza A virus and lymphocytic choriomeningitis virus (LCMV), and analyzing lung or splenic transcriptomes, respectively, gave important insights into the systems regulation. Altboum et al. developed a computational method named digital cell quantifier (DCQ) that infers quantitative changes of over 200 defined immune cell subpopulations from time-resolved lung transcriptome data (11). They then predicted and subsequently verified experimentally that different dendritic cell populations have specific roles at early and late time points of acute Flu infections. More recently, we used weighted gene coexpression network analysis (WGCNA) to characterize systems perturbations during acute and chronic LCMV infections (1). From spleen transcriptome-derived coexpression modules and subsequent immunological analyses we demonstrated a delicate adaptation process toward a chronic virus infection with both immunosuppressive and immunostimulatory processes involved. However, only a tiny fraction of the global information content has been utilized and the bulk awaits exploitation.

To gain better insights into the mechanisms of chronicity development and virus control, we here combined DCQ with WGCNA, and explored further our time-resolved splenic transcriptome data sets. We show that: (i) DCQ predictions fit well with the current knowledge of the immune cell dynamics during acute and chronic LCMV-infections, (ii) the combination of WGCNA and DCQ allows to better characterize the dynamic cell events occurring in complex tissues, and (iii) during the evolution toward the chronic infection state, the chemokine XCL1 is produced by CD8^+^ T cells that express markers of early effector cells. Together this demonstrates the utility of combining DCQ and WGCNA for analyzing complex RNA-Seq data sets.

## Materials and Methods

### Animals, Infections, and Depletion of Macrophages

Male C57BL/6J mice aged 4–8 weeks were purchased from Charles River Laboratories and maintained under specific pathogen-free conditions at the animal facility of the Parc de Recerca Biomèdica de Barcelona (PRBB). Animals were treated according to the Guidelines of the Basel Declaration and from the Generalitat de Catalunya (project number 9422), and approved by the ethical committee for animal experimentation (CEEA-PRBB, Spain; permit license number JMR-16-0046). Animals were infected intraperitoneally (i.p.) with either 2 × 10^2^ (low dose, LD) or 2 × 10^6^ (high dose, HD) plaque forming units (PFU) of the strain Docile of LCMV (4–7 animals per group) to induce an acute or chronic infection, respectively. Viral titers from spleens of infected mice were determined on MC57 cells using focus-forming assay (12). For *in vivo* depletion of macrophages, mice were injected i.v. with 300 μl of clodronate-loaded liposomes (Liposoma BV; 5 mg/ml) (13), or PBS-loaded liposomes as a control.

### Cell Surface and Intracellular Cytokine Staining by Flow Cytometry

For Flow Cytometry analysis and cell sorting, spleens were harvested and single-cell suspensions were generated. Cells were then stained with the following antibodies to analyze B cells, and effector and regulatory T cells: CD4-PE (Clone H129.19), CD8-PECy5 (Clone 53-6.7), CD8a-PercpCy5.5 (Clone 53-6.7), CD25-APCCy7 (Clone PC61), CXCR5-PECy7 (Clone SPRCL5), CD83-Alexa Fluor 488 (Clone Michel-19), CD199-BV421 (Clone CW-1.2), CD153-BV421 (Clone RM153), CD19-FITC (Clone 1D3), CD43-PE (Clone eBioR2/60), CD5-APC (Clone 53-7.3), IgM-PECy7 (Clone II/41), CD23-eFluor450 (Clone B3B4), XCL1-Unconjugated (Clone 80222), mouse anti-rat IgG2a-Alexa Fluor 647 (Clone 2A8F4), IFNɤ-FITC (Clone XMG1.2), FOXP3-Alexa Fluor 647 (Clone MF23), and the polyclonal TLR7-FITC. To analyze monocyte/macrophage and neutrophil populations, cells were stained with CD3e-PECy7 (Clone 145-2C11), NK1.1-PECF594 (Clone PK136), CD11b-APC (Clone M1/70), and CD27-FITC (Clone LG.7F9) for natural killer T cells, and with CD45R-PECF594 (Clone RA3-6B2), NK1.1-PECF594 (Clone PK136), CD11c-PercpCy5.5 (Clone HL3), CD11b-PECy7 (Clone M1/70), Ly-6G-PE (Clone 1A8), and Ly-6C-FITC (Clone AL-21). For determination of XCL1- and TLR7-producing T cells, splenocytes were directly put into media containing Brefeldin A (Sigma Aldrich) without stimulation before intracellular cytokine staining (ICS). Staining of FOXP3-expressing cells was performed following the manufacturer instructions (eBiosciences). To visualize IFN production, cells were first stimulated with LCMV gp33 peptide for 3 h followed with the addition of brefeldin A for 2 h. All antibodies were purchased from either BD Biosciences, eBioscience, Biolegend or R&D Systems. A LSR Fortessa (BD Biosciences) was used for flow cytometry and data were analyzed using FlowJo 10.1 software. A FACSAria II SORP (BD Biosciences) sorter was used for cell sorting. All samples were kept at 4°C during cell sorting. Sort purity was >95% for all cell populations.

### Digital Cell Quantifier (DCQ)

DCQ was performed as previously described (11). Briefly, the DCQ took as an input: (i) an immune cell compendium of transcriptional profiles, consisting of 213 different immune cell subsets and their corresponding cell surface markers; and (ii) differentially expressed genes from spleens from acute and chronic LCMV-infected mice (1). We used the glmnet R package (14) with the parameters α = 0.05, lambda.min.ratio = 0.2. To evaluate the robustness of the predicted results, DCQ was run 100 times using only a random collection of 50% of the cell types in the compendium on each run, resulting in 100 different solutions. Standard deviations were calculated across these 100 solutions. The robustness score (significance of a predicted change in quantity) was assessed by evaluating whether the sample of relative quantities is significantly different from zero (*p*-value score). Significantly changing cell types were defined as those whose –Log_10_ *p*-value score was lower than −20 (cell decrease) or higher than 20 (cell increase) in at least one of the infections (Supplementary Table 1).

### ImmGen Data

To compare gene expression levels between early and late effector CD8^+^ T cells, the tool “Population Comparison” from the ImmGen data browser (http://www.immgen.org/) was used. This tool provided a ranked table of genes that are always expressed in OVA-specific effector CD8^+^ T cells analyzed 12 and 24 h post-infection with Listeria (LisOva) and never expressed in the same cells analyzed at days 5, 6, and 8 post-infection with Listeria (LisOva) or Vesicular stomatitis virus (VSVOva). Default thresholds from the ImmGen tool were used. To analyze the pattern of expression of the genes *Xcl1, Tnfsf8, Tlr7, Ccr9*, and *Cd83* across OVA-specific CD8^+^ T cells in the ImmGen compendium, we used the tool “My GeneSet” (http://www.immgen.org/) using Microarray V1 data set. Expression values were obtained as the log_2_ of each gene expression value/average expression value of all genes.

### RNA-Sequencing and Bioinformatic Analysis

Total RNA from sorted cells from uninfected (2 pools of 2 mice, day 0) or acute (2 pools of 2 mice, day 0, day 7) infected mice (5 × 10^4^ cells per sample) was isolated according to the manufacturer's instructions using Qiagen RNeasy Micro kit (Qiagen). The quality and concentration of RNA were determined by an Agilent Bioanalyzer. RNA was submitted for sequencing to Macrogen Inc. (Seoul, Korea). Sequencing libraries were obtained after removing ribosomal RNA by a Ribo-Zero kit (Illumina). cDNA was synthesized and tagged by addition of barcoded Truseq adapters. Libraries were quantified using the KAPA Library Quantification Kit (KapaBiosystems) prior to amplification with Illumina's cBot. Four libraries were pooled and sequenced (single strand, 50 nts) on an Illumina HiSeq2000 sequencer to obtain 50–60 million reads per sample. RNA-Seq reads were mapped to the reference mouse genome (GRCm38, gencode M18) with STAR (15) and genes were quantified with RSEM (16). Differential expression analysis was performed with DESeq2 (17). Genes with a false discovery rate (FDR) <5% were considered significant. Gene ontology (GO) enrichment analysis was performed with DAVID (http://david.ncifcrf.gov/) (18).

### Statistical Analysis

Statistical analyses were performed using GraphPad Prism software version 6.0 (GraphPad Software Inc., CA, USA). Data were analyzed using non-parametric one-way ANOVA or two-tailed *t-*test. For correlation between modules and DCQ-inferred cell kinetics, Pearson's correlation was used. Fisher's exact test was used to quantify the significance of gene overlap between acute-brown module hub genes and genes from CD8^+^ T cells and monocytes/macrophage cell subsets. Non-significant differences were indicated as “ns.” *P*-values (p) below 0.05 were considered significant and were indicated by asterisks: ^*^p ≤ 0.05; ^**^p ≤ 0.01; ^***^p ≤ 0.001; ^****^p ≤ 0.0001.

### Data Access

The complete RNA-Seq datasets are available from the Gene Expression Omnibus (accession number GSE123134).

## Results

### Immune Cell Dynamics During Acute and Chronic LCMV Infection

**Figure 1:**
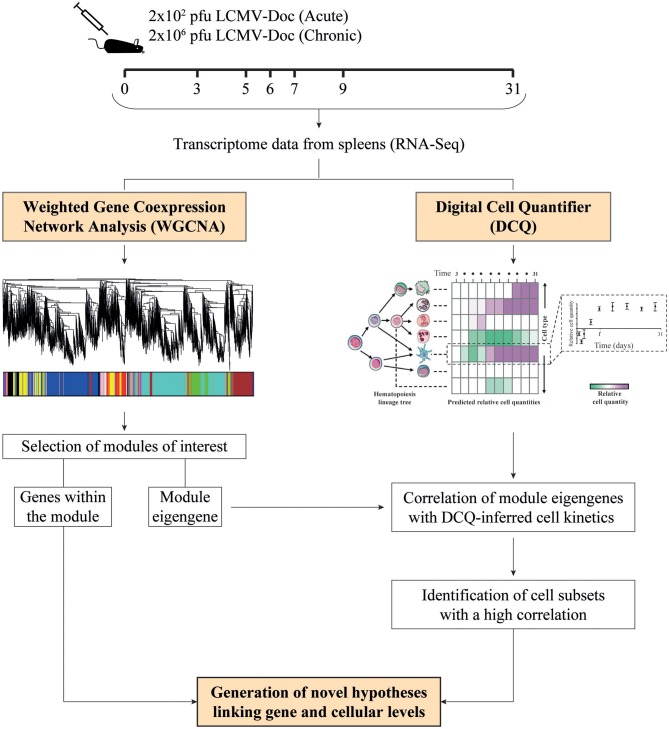
Schematic representation of the experimental design. Mice were infected with either 2 × 10^2^ or 2 × 10^6^ PFU of LCMV Docile (LCMV_Doc_), and spleens were collected at the indicated days post-infection to obtain time-resolved transcriptomes in acute and chronic infections, respectively. Differentially expressed (DE) gene kinetics obtained by RNA-Seq were used as input for weighted gene coexpression network analysis (WGCNA) and digital cell quantifier (DCQ) to obtain modules of highly coexpressed genes and predictions of immune cell kinetics in spleen, respectively. To identify the cell subsets expressing a cluster of genes from a particular module, we performed a Pearson's correlation analysis of the module eigengene with DCQ-inferred cell kinetics, and novel hypotheses are generated.

To obtain a global view of the biological processes that participate in and control acute and chronic LCMV infection fates, we created a new computational approach that combines WGCNA-derived gene coexpression networks with DCQ-inferred immune cell kinetics. As input, we used our previously generated RNA-Seq data set (1) that consists of time-resolved splenic transcriptomes from C57BL/6J mice infected with a low-dose (2 × 10^2^ PFU; acute infection) or a high-dose (2 × 10^6^ PFU; chronic infection) of LCMV strain Docile (LCMVDoc) (Figure 1). The time points [days 0, 3, 5, 6, 7, 9, and 31 post-infection (p.i.)] were selected according to the main viral and immunological features, and therefore represent the main states of an acute and a chronic LCMV infection. Thirteen thousand nine hundred seventy-one genes were identified as differentially expressed (DE) when compared to uninfected animals, and were analyzed by WGCNA to obtain modules of highly coexpressed genes (1) (Figure 1).

**Figure 2:**
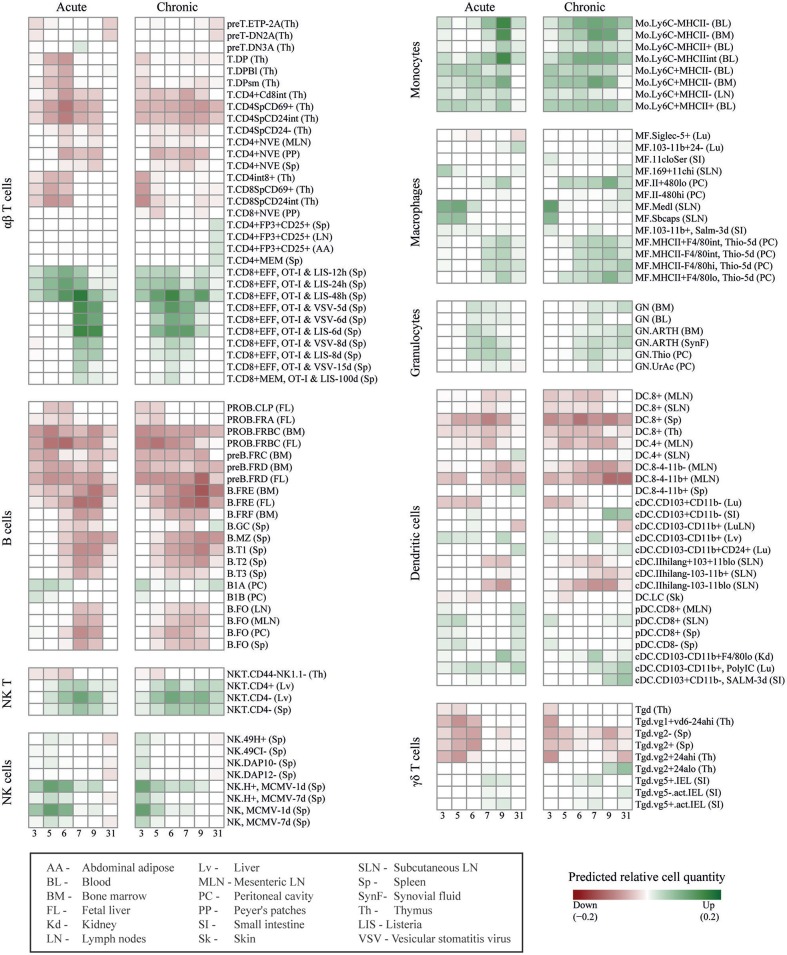
DCQ reconstruction of global immune cell dynamics during acute and chronic LCMV infections. Global dynamics in immune cell quantities (green/increase, red/decrease in relative cell quantities) after acute and chronic LCMV infections, predicted by DCQ at different time points post-infection (columns) for 125 different immune cell types (rows). Each cell type heading is followed by the code of the tissue from which the cell type was isolated in the compendium. For effector CD8^+^ T cells cells obtained from infected mice, pathogens, and time points post-infection are also indicated. The box at the bottom left contains details for these abbreviations.

To predict the immune cell dynamics during acute and chronic LCMV infection, we used the expression kinetics of the DE genes as an input for DCQ (11). The DCQ output consisted of the suggested kinetics of 207 different immune cell subsets. Of these, 125 cell subsets had a significant change in their quantity between at least two consecutive time points (robustness score higher/lower than ± 20, see methods). A comprehensive map of the dynamic changes of these 125 cell subsets during the infection courses is shown in Figure 2. Sixty-eight cell subsets were predicted to increase and 57 were predicted to decrease in both, acute and chronic infection (Supplementary Table 1). Note that the different cell subsets are named according to the nomenclature of the immune cell compendium which was used to establish DCQ (11). The respective names are also used below and given in brackets when referring to the different subsets as of Figure 2.

Effector CD8^+^ T cells play a critical role during LCMV infections. They control virus expansion in acute infection while CD8^+^ T cell exhaustion is a hallmark of chronic infection (19). After an acute LCMV_Doc_ infection, virus-specific CD8^+^ T cells expand at d6-d7 and their percentages remained high at d31. In contrast, during a chronic infection, IFN ɤ-producing CD8^+^ T cells drop in their numbers at d7-d9 (Supplementary Figure 1A), and maintain an elevated expression of inhibitory receptors such as PD-1 and TIM-3 (1). In order to verify that DCQ can correctly predict changes in the dynamics of immune cell subsets with major roles during LCMV infection, we first focused on the DCQ-inferred kinetics of effector CD8^+^ T cells. The original compendium of immune cells used as input for the DCQ contains effector CD8^+^ T cells obtained at 5, 6, and 8 days post-infection with Listeria (T.CD8+EFF, OT-I & LIS) or Vesicular stomatitis virus (T.CD8+EFF, OT-I & VSV). DCQ correctly predicted an increase of these effector CD8+ T cells in both acute and chronic LCMV infections (Figure 2 and Supplementary Figures 1A,B). Importantly, DCQ also predicted exhaustion in chronic infection, showing a drastic decrease of these effector cells between days 7 and 9 post-infection with LCMV (Supplementary Figure 1B). Moreover, the kinetics of memory CD8^+^ T cells (T.CD8+EFF, OT-I & VSV-15 days, and T.CD8+MEM, OT-I & LIS-100 days) also showed the failure of chronically infected mice to generate a memory T cell response, in contrast to acute infected mice (Figure 2 and Supplementary Figure 1B).

DCQ also correctly predicted the changes in immune cell quantities of several other cell subsets with a specific role in chronic LCMV infection. For example, CD4^+^ regulatory T cells (T.CD4+FP3+CD25+) only increased late in chronic infection (day 31 p.i.), as previously described (20) and further validated by flow cytometry (Supplementary Figure 2A). Two subsets of conventional dendritic cell (cDC) expressing the marker CD103 showed an increase from day 9 in chronic infection (cDC.CD103+CD11b–) (Supplementary Figure 2B). These CD103^+^ CD11b^−^ DCs that were sampled for the immune cell compendium from intestine, are also present in other tissues such as the spleen. They express CD8 and the chemokine receptor XCR1, and are specialized in antigen cross-presentation (21). Thus, the predicted DC kinetics likely represents the appearance of XCR1^+^ DCs that we have recently described to contribute to the maintenance of an antiviral cytotoxic T cell response and viral control during the chronic infection phase (1). Finally, DCQ also predicted a transient increase of two neutrophil subsets (GN.ARTH) in acute infection that only in chronically infected mice remained elevated at day 31 p.i. (Supplementary Figure 2C). These neutrophils, which were monitored from arthritic mice in the immune cell compendium, likely represent the previously reported appearance of neutrophilic suppressor cells which have an immunomodulatory role during chronic infections (22).

Other predicted immune cell subset kinetics showed a similar overall behavior in acute and chronic infected mice. For example, despite previous reports that attributed different roles to NK cells in the two infection outcomes (23, 24), activated NK cells (NK and NK.H+, MCMV) showed a similar kinetic in acute and chronic infection (Figure 2 and Supplementary Figure 3A), with an early peak at days 5 and 3 p.i., respectively. The predicted increase of activated NK cells was validated by analyzing the kinetics of NK cells at different maturation states by staining cells with anti-CD11b and anti-CD27 antibodies (25). Interestingly, only activated effector NK cells coexpressing these two surface marker showed the kinetics as predicted by DCQ while immature NK cells differed (Supplementary Figure 3B). This nicely demonstrates the ability of DCQ to predict the quantity of immune cell subsets in a particular functional state. DCQ-predictions of monocyte kinetics were also validated by flow cytometry, showing a rapid increase of inflammatory Ly6c^+^ monocytes followed by an increase of resident Ly6c^−^ monocytes at later time points in both acute and chronic infection (Supplementary Figures 3C,D). Finally, B cells showed a decrease in numbers in both acute and chronic infections (Figure 2 and Supplementary Figure 4), in agreement with previous publications that reported a type I IFN- or NK-mediated depetion of B cells in LCMV infection (26–28).

### WGCNA-Derived Modules Representing T Cell Responses Correlate With Effector CD8^+^ T Cells and Macrophages

**Figure 3:**
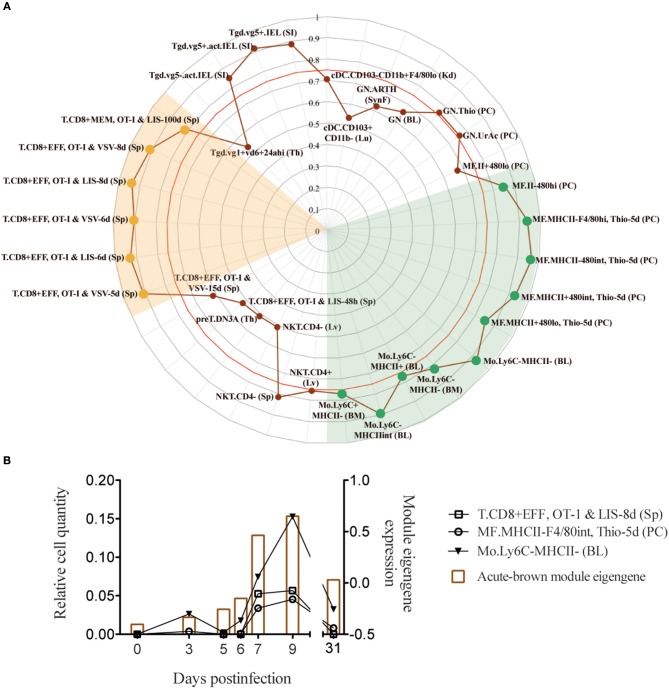
Acute-brown module correlates with effector CD8^+^ T cells and monocyte/macrophage subsets. **(A)** Radar chart showing the correlation values of immune cell subsets from acute infection with acute-brown module eigengene kinetics (only shown cell subsets with a correlation >0.5). Red line shows Pearson's correlation score with a *p* = 0.05. **(B)** Kinetics of acute-brown module eigengene (right axis) and the CD8^+^ T cell, macrophage and monocyte subsets (left axis) with the highest correlation scores.

**Figure 4:**
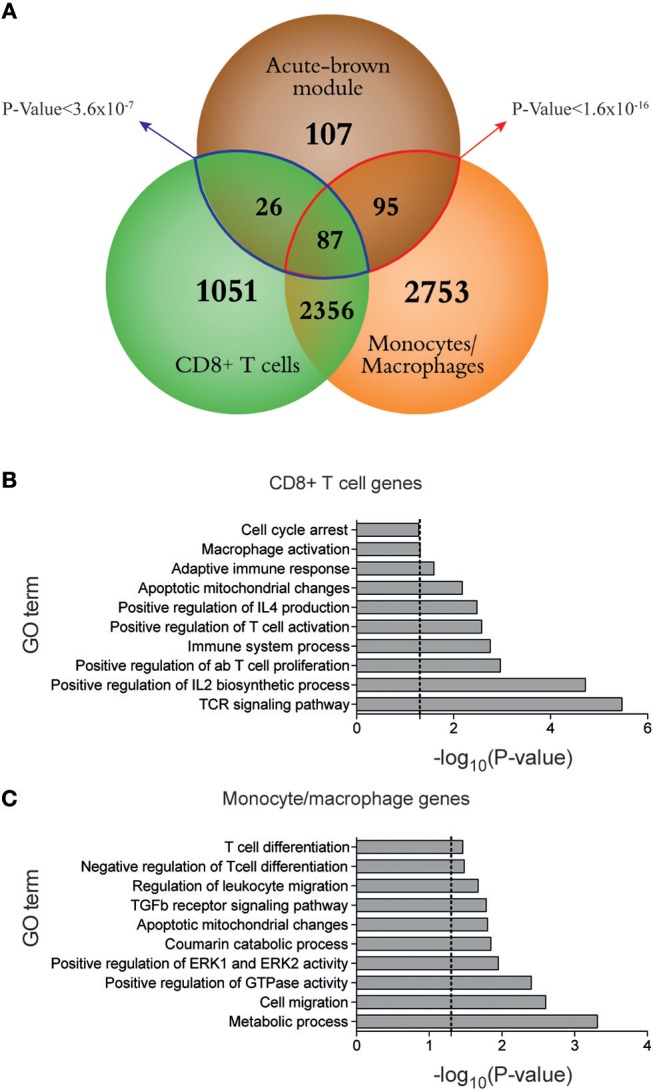
Monocytes/macrophages and CD8^+^ T cells cooperate in the induction of the T cell response during LCMV acute infection. **(A)** Venn diagram of overlaps among differentially expressed genes between days 0 and 7 after acute LCMV infection from sorted monocytes/macrophages and CD8^+^ T cells, and hub genes from acute-brown module [genes with intramodular connectivity (K_IM_)>0.6]. The significance of gene overlap between monocytes/macrophages (red line) and CD8^+^ T cells (blue line) with acute-brown module was calculated by Fisher's Exact Test. **(B–C)** Ten representative GO terms enriched in genes from CD8^+^ T cells **(B)** and monocytes/macrophages **(C)** (dashed lines mark *p* = 0.05). Enrichment analysis was performed using DAVID (http://david.ncifcrf.gov/).

**Figure 5:**
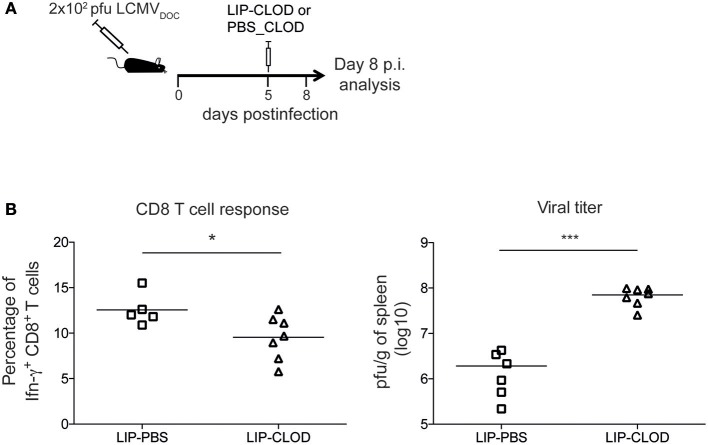
Macrophages contribute to the induction of CD8^+^ T cells response during acute LCMV infection. **(A)** Schematic outline of clodronate liposome treatment for depletion of macrophages. **(B)** Percentages of IFNɤ-producing CD8^+^ T cells and virus titers in spleen from acute infected mice treated (LIP-CLOD) or non-treated (LIP-PBS) with clodronate liposomes at day 8 p.i. Significant differences were determined by one-way ANOVA. ^*^p ≤ 0.05; ^***^p ≤ 0.001.

Using the same RNA-Seq data set from acute and chronic LCMV infections, our group previously generated spleen transcriptome-derived coexpression modules by WGCNA (1). This analysis provided relevant information about biological processes playing a major role in response to virus-induced host perturbations. However, the gene coexpression analysis of total organ RNA did not provide information about the cell subsets that participate in the expression of the genes within the coexpression modules. In order to decipher which immune cell subsets are involved in spleen-derived gene coexpression modules, we hypothesized that, in some circumstances, the kinetics of a set of coexpressed genes will correlate with the kinetics of the cell subsets expressing them. On this basis, we performed a Pearson's correlation analysis between WGCNA-derived module eigengenes (1) and DCQ-inferred cell kinetics (Figure 1). We first analyzed which DCQ-inferred immune cell subset kinetics from acute infection correlated with the acute-brown module eigengene. This module was previously identified as the representative of the LCMV-specific CD8^+^ T cell response induced in acute infection. Its eigengene expression kinetics highly correlated with the LCMV-specific CD8^+^ T cell response and the 315 hub genes within the module revealed an enrichment for T cell activation genes (1). Twenty-three out of the one hundred twenty-five immune cell subsets inferred by DCQ showed a significant positive correlation (*p* < 0.05) with the acute-brown module eigengene (Supplementary Table 2). As expected, effector CD8^+^ T cells (T.CD8+EFF, OT-I; monitored at days 5, 6, and 8 post-infection with LIS or VSV) showed correlation scores above 0.9 (Figure 3A and Supplementary Table 2), thus indicating that our approach correctly predicts the immune cell subsets responsible for the expression of genes within the module. Interestingly, several monocyte and macrophage cell subsets also showed high correlation scores (Figures 3A,B). To test whether these cells subtypes also express genes contained in the acute-brown module, we performed RNA-Seq analyses of sorted monocytes/macrophages and CD8^+^ T cells from naive mice, and animals infected with a low dose of LCMV_Doc_ (acute infection). A total of 5291 genes were significantly upregulated at day 7 p.i. in activated CD44^+^ CD8^+^ T cells compared to CD44^−^ CD8^+^ T cells from uninfected naive mice. Monocytes/macrophages showed 3,520 genes significantly upregulated at day 7 p.i. compared to the cells from uninfected mice. To analyze whether the genes within the acute-brown module were significantly enriched for genes from these two cell subsets, we determined the gene overlap between the module hub genes and the genes upregulated in CD8^+^ T cells and monocytes/macrophages by a Fisher's Exact Test (Figure 4A). The acute-brown module was highly enriched for genes upregulated in both cell subsets. From the 315 hub genes within the module, 113 overlapped with genes upregulated in activated CD8^+^ T cells (*p* < 3.6 × 10^−7^) and were enriched for genes involved in the processes of TCR signaling pathway, T cell activation and IL4 production (Figure 4B and Supplementary Table 3A). Importantly, 182 hub genes overlapped with genes upregulated in monocytes/macrophages (*p* < 1.6 × 10^−16^) and were enriched for genes involved in T cell response, TGF-β signaling and leukocyte migration, among others (Figure 4C and Supplementary Table 3B), thus indicating that the acute-brown module represents the complex process of induction of the adaptive T cell response that requires the coordination of monocytes/macrophages and CD8^+^ T cells. To validate this hypothesis further, we analyzed CD8^+^ T cell response and virus loads in spleens from acutely infected mice after depletion of macrophages (Figure 5A). Mice treated with clodronate liposomes showed a significant decrease in percentages of IFNɤ-producing cells and an increase of virus loads at day 8 p.i. (Figure 5B), thus demonstrating that macrophages contribute to the induction of the T cell response. All together, these results demonstrate that the combination of DCQ and WGCNA is a very valuable tool to better characterize immune cell subsets that participate in a complex biological pathway represented by a gene coexpression module.

### XCL1-Producing Cells During the Adaptation Phase to a Chronic Infection Present an Immature Early Effector Phenotype

**Figure 6:**
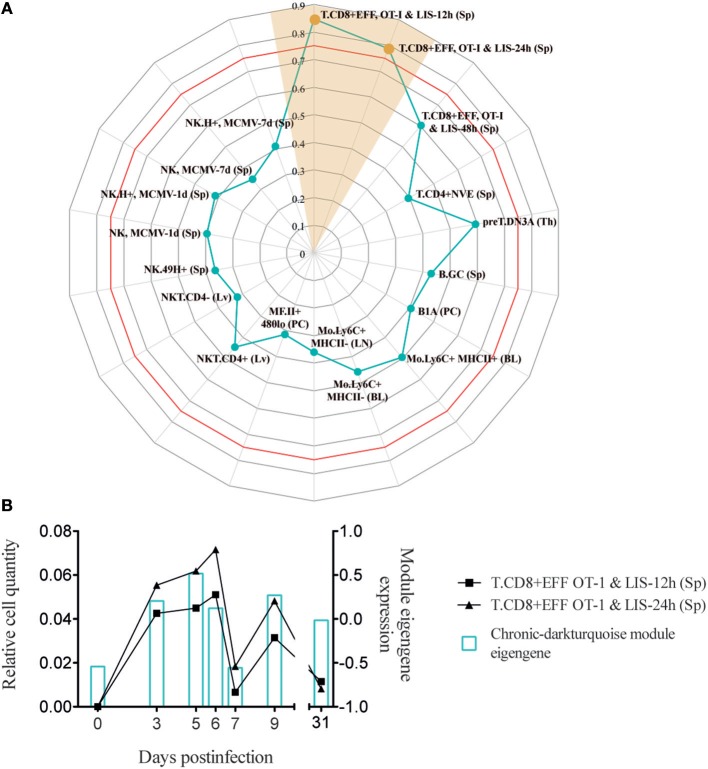
Chronic-darkturquoise module correlates with early effector CD8^+^ T cells. **(A)** Radar chart showing the correlation values of immune cell subsets with chronic-darkturquoise module eigengene kinetics (only shown cell subsets with a correlation >0.3). Red line shows the Pearson's correlation score with a *p* = 0.05. **(B)** Kinetics of early effector CD8^+^ T cells (left axis) and chronic-darkturquoise module eigengene (right axis).

Analysis of infection-fate-specific modules in Argilaguet et al. (1) allowed to identify the biological pathways specific of chronic infection. In particular, the chronic-darkturquoise module contains the chemokine XCL1, showing a “two-peak” behavior with an expression peak at day 5 and a second upregulation from day 7 to day 9 p.i., at the time when exhaustion of CD8^+^ T cells appears (Figure 6B and Supplementary Figure 1A). We showed that XCL1 expression resulted in the recruitment of cross-presenting dendritic cells that express the XCL1 receptor XCR1, and that these dendritic cells contributed to the maintenance of the antiviral cytotoxic T cell response and viral control in the chronic infection phase. XCL1 was mainly produced by LCMV-specific CD8^+^ T cells expressing CXCR5, a marker of exhausted CD8^+^ T cells that retain effector functions (29, 30). However, due to the complexity of effector and exhausted CD8^+^ T cell subpopulations present during a chronic infection (4), a detailed phenotypic characterization of XCL1-producing CD8^+^ T cells was lacking.

**Figure 7:**
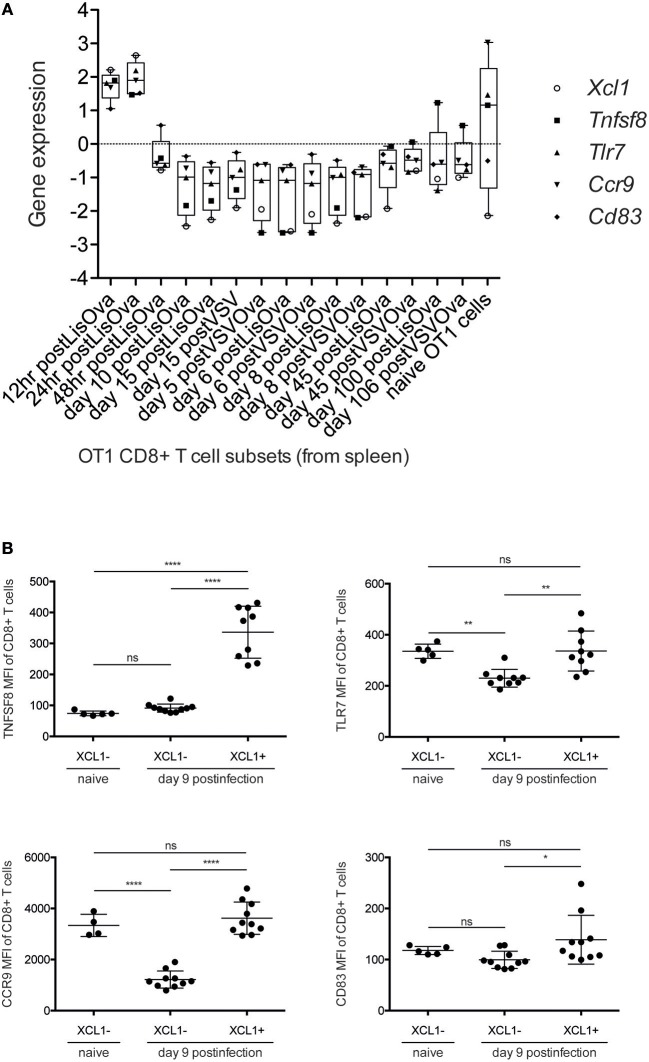
XCL1-producing CD8^+^ T cells express markers characteristic of early effector CD8^+^ T cells. **(A)** Box-plot representation of *Xcl1, Tnfsf8, Tlr7, Ccr9, and Cd83* expression. Mean-normalized expression values (Log2) of selected genes in Ova-specific OT1 CD8^+^ T cells were obtained using “My GeneSet” tool from ImmGen (http://www.immgen.org/). **(B)** Mean fluorescence intensity (MFI) of *Tnfsf8, Tlr7, Ccr9, and Cd83* in XCL1^−^ and XCL1^+^ CD8^+^ T cells in naive mice and in chronically-infected animals at day 9 p.i. Significant differences were determined by one-way ANOVA. ns, non-significant; ^*^p ≤ 0.05; ^**^p ≤ 0.01; ^****^p ≤ 0.0001.

In order to better characterize the phenotype and activation state of XCL1-producing CD8^+^ T cells in chronic LCMV infection, we used our approach to analyze which DCQ-inferred immune cell subset kinetics from chronic infection correlated with the chronic-darkturquoise module eigengene. Interestingly, only CD8^+^ T cell subsets isolated at 12 and 24 h post-Listeria infection (T.CD8+EFF, OT-I & LIS-12 and 24 h) showed a significant correlation (Figure 6 and Supplementary Table 2), suggesting that XCL1-producing CD8^+^ T cells have an immature early effector phenotype. Using the “Population Comparison” tool from the Immunological Genome (ImmGen) Project, we next analyzed which genes are upregulated in these two cell subsets compared to late effector CD8^+^ T cells (T.CD8+EFF, OT-I; monitored at days 5, 6, and 8 post-infection with LIS and VSV; see methods). Three hundred eighty-five genes showed expression values significantly higher in early vs. late effector CD8^+^ T cells. To note, within them we found XCL1 and CXCR5, with a mean fold-change of 25.14 and 4.94, respectively, further indicating that XCL1 is produced by CD8^+^ T cells with a phenotype characteristic of immature early effector CD8^+^ T cells. To validate this hypothesis further, we analyzed protein expression characteristic for early effector CD8^+^ T cells in chronically infected mice at day 9 by flow cytometry. Using the “My GeneSet” tool from ImmGen, we selected four proteins with high gene expression values in early effector T cells: TNFSF8, a cytokine that induces proliferation of T cells and that has been shown to be upregulated in CD8^+^ T cells primed during chronic infection (31); TLR7, a receptor selectively upregulated by exhausted CXCR5^+^ CD8^+^ T cells (29); CCR9, a chemokine receptor that regulates early phases of inflammation (32); and CD83, which is upregulated upon T cell stimulation during virus infection (33) (Figure 7A). Expression of TNFSF8, TLR7 and CD83 by XCL1-negative CD8^+^ T cells was similar between naive mice and infected animals at day 9 p.i. (Figure 7B). By contrast, CCR9 showed higher expression levels in naive mice, in concordance with data from the ImmGen dataset, in which CCR9 showed a high gene expression value in naive OT1 cells (Figure 7A). Importantly, TNFSF8, TLR7 and CCR9 were highly expressed by XCL1^+^ CD8^+^ T cells at day 9 p.i., showing mean fluorescence intensities (MFI) significantly higher than that in XCL1^−^ cells (Figure 7B). These results demonstrate that during the adaptation phase to a chronic infection, XCL1 is produced by CD8^+^ T cells with characteristic early effector cell marker. All together, our results demonstrate that by combining WGCNA with DCQ, it is possible to define cell—cell cooperativity as well as the activation and differentiation status of cells participating in the immune response to virus infections.

## Discussion

Here we describe a versatile computational approach that combines WGCNA-derived gene coexpression networks with DCQ-inferred immune cell kinetics. It enables to generate hypotheses about complex immune system functioning after a virus-induced perturbation. We show, first, the ability of DCQ to predict changes of immune cell subsets that play major roles during an LCMV infection. Second, we characterize immune cell subsets involved in spleen-derived gene coexpression modules that cooperate in complex biological pathways. Finally, we predict and subsequently verify experimentally that virus-specific CD8^+^ T cells in the chronic infection phase resemble early effector cells.

To derive information about the immune system functioning after a perturbation or under pathological conditions solely from transcriptome data of whole tissue specimen is challenging. Algorithms like DCQ (11), seq-ImmuCC (34), or CoD (35) can predict dynamic quantities of cell subtypes, however they fail to distinguish between immunological mechanisms like cell migration or cell differentiation. Gene coexpression analysis like WGCNA, on the other hand, can order the several thousand genes from RNA-Seq runs according to common dynamic features however cannot predict cell types. With the combination of both, hypothesis generation becomes easier and goes beyond what both methods can provide by their own. For example, we could identify macrophages as an important immune cell type that cooperates with CD8^+^ T cells in the induction of an adaptive immune response (Figures 4, 5). It is known that dendritic cells are not critical for CD8^+^ T cell priming in LCMV (36). Moreover, they decline in numbers shortly after infection (Figure 2) (22). Since other antigen presenting cells like macrophages and B cells are also infected by LCMV (37–39), and can efficiently present virus-derived peptide in conjunction with MHC-I proteins on their surface, they seem to contribute to the activation of the CTL response and thereby participate in the control of virus expansion. Furthermore, we were able to characterize an important immunological event during chronic infection that appear concomitant with exhaustion. Indeed, we demonstrate that XCL1-producing CD8^+^ T cells have an early effector phenotype and differ fundamentally from the effector cells during acute infections. This observation is a step forward in our understanding of the immune adaptation process to a chronic infection. However, further work is necessary to decipher whether these cells emerge from “*de novo*” primed naive CD8^+^ T cells or from exhausted cells that “recover” an effector functional state. Thus, by combining WCGNA with DCQ one can identify cell cooperativity and specify cellular phenotypes participating in critical biological events during perturbations.

The novel combination of WGCNA and DCQ for interpreting time-resolved transcriptome data from acute and chronic virus infections is a step forward for our understanding of the complex immune responses during pathogen invasion. However, it is just a tiny part of the overall process that needs to be complemented with other measures like *in situ* imaging techniques, single cell analyses and whole organism studies (2, 6, 40). Furthermore, computational approaches will be necessary to integrate all available data and generate hypotheses about the underlying regulatory principles that make the highly complex, diverse and dynamic immune system so functionally robust against pathogens. While many of the required technologies are in place, the data integration will require tight collaborations across disciplines engaging biologists, clinicians, physicists, and mathematical modelers. With this, one can easily envision predictive frameworks that will help in the rational design of therapies in infectious diseases and cancers. An exciting time lies ahead.

## Ethics Statement

This study was carried out in accordance with the recommendations of the Guidelines of the Basel Declaration and from the Generalitat de Catalunya (project number 9422). The protocol was approved by the ethical committee for animal experimentation (CEEA-PRBB, Spain; permit license number JMR-16-0046).

## Author Contributions

JA and AM designed the study. MP, GR, VC, and CS performed experiments. MP, AE-C, YS, SH, and IG-V performed the bioinformatic analyses of the RNA-Seq data sets including WGCNA and DCQ. MP and JA accessed the bioinformatic data output. JA, MP, GB, and AM interpreted the data and wrote the manuscript. All authors read and approved the final manuscript.

### Conflict of Interest Statement

The authors declare that the research was conducted in the absence of any commercial or financial relationships that could be construed as a potential conflict of interest.
