# Supplementary material for: Linking Cell Dynamics With Gene Coexpression Networks to Characterize Key Events in Chronic Virus Infections
Source: Front Immunol. 2019 May 3;10:1002. doi: 10.3389/fimmu.2019.01002 (PMC6509617; doi:10.3389/fimmu.2019.01002)
Supplement: Supplementary file 4 [file Data_Sheet_1.PDF]

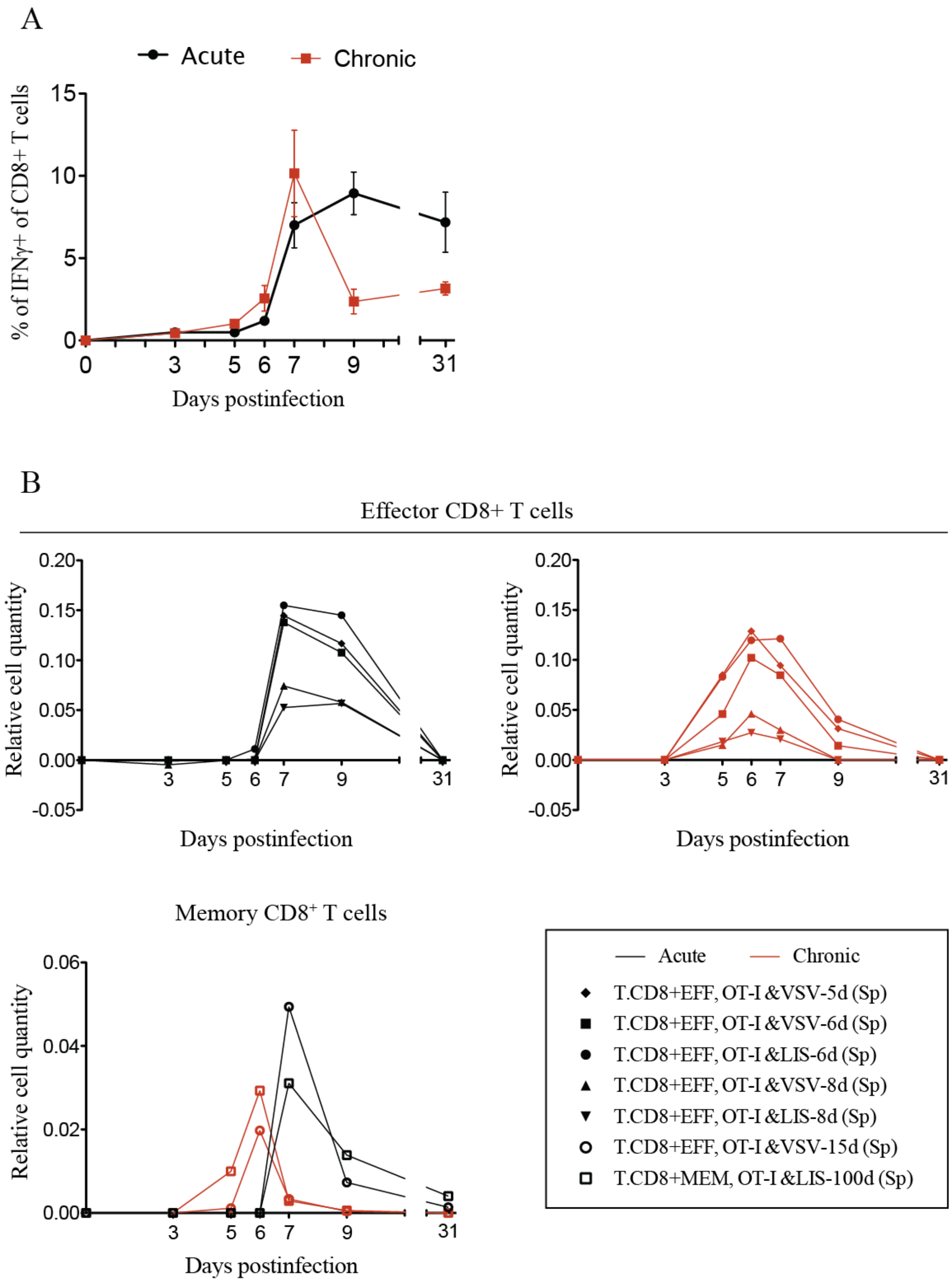

Supplementary Figure 1. Dynamics of effector CD8$^+$ T cells predicted by DCQ show exhaustion appearance in chronic LCMV infection. (A) Percentages of GP33-specific IFNγ-producing CD8$^+$ T cells in spleen. The mean ± SEM is shown. (B) DCQ-inferred cell kinetics of effector CD8$^+$ T cells isolated after VSV or Listeria infections at the indicated time points.

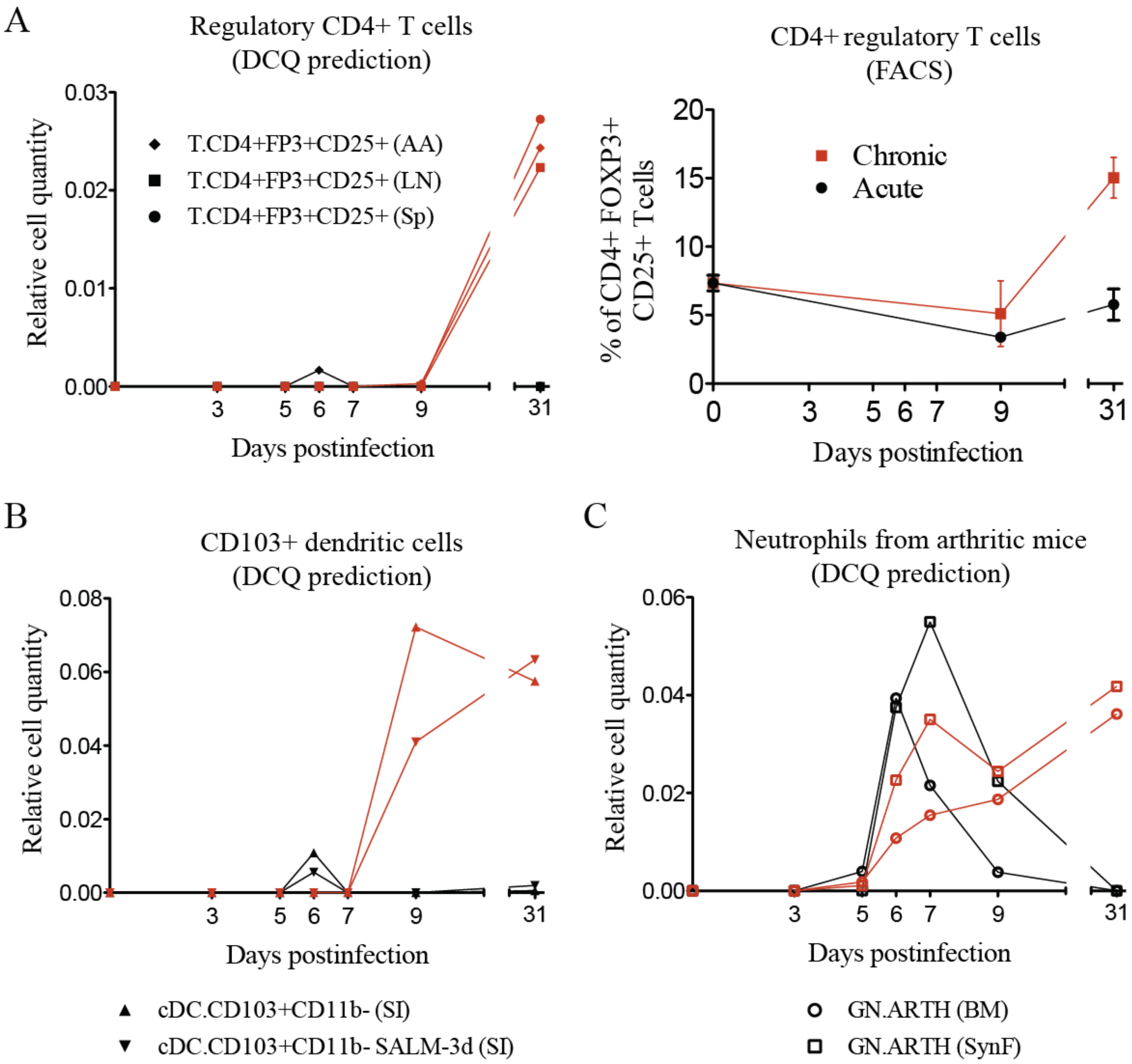

Supplementary Figure 2. DCQ-predictions of immune cell subsets with specific roles during chronic infection. (A) DCQ cell predictions and validation by FACS of regulatory $CD4^+$ $Foxp3^+$ T cells. (B-C) DCQ cell predictions of $CD103^+$ dendritic cell subsets (B) and neutrophils from arthritic mice (C) from acutely (black lines) or chronically (red lines) infected mice.

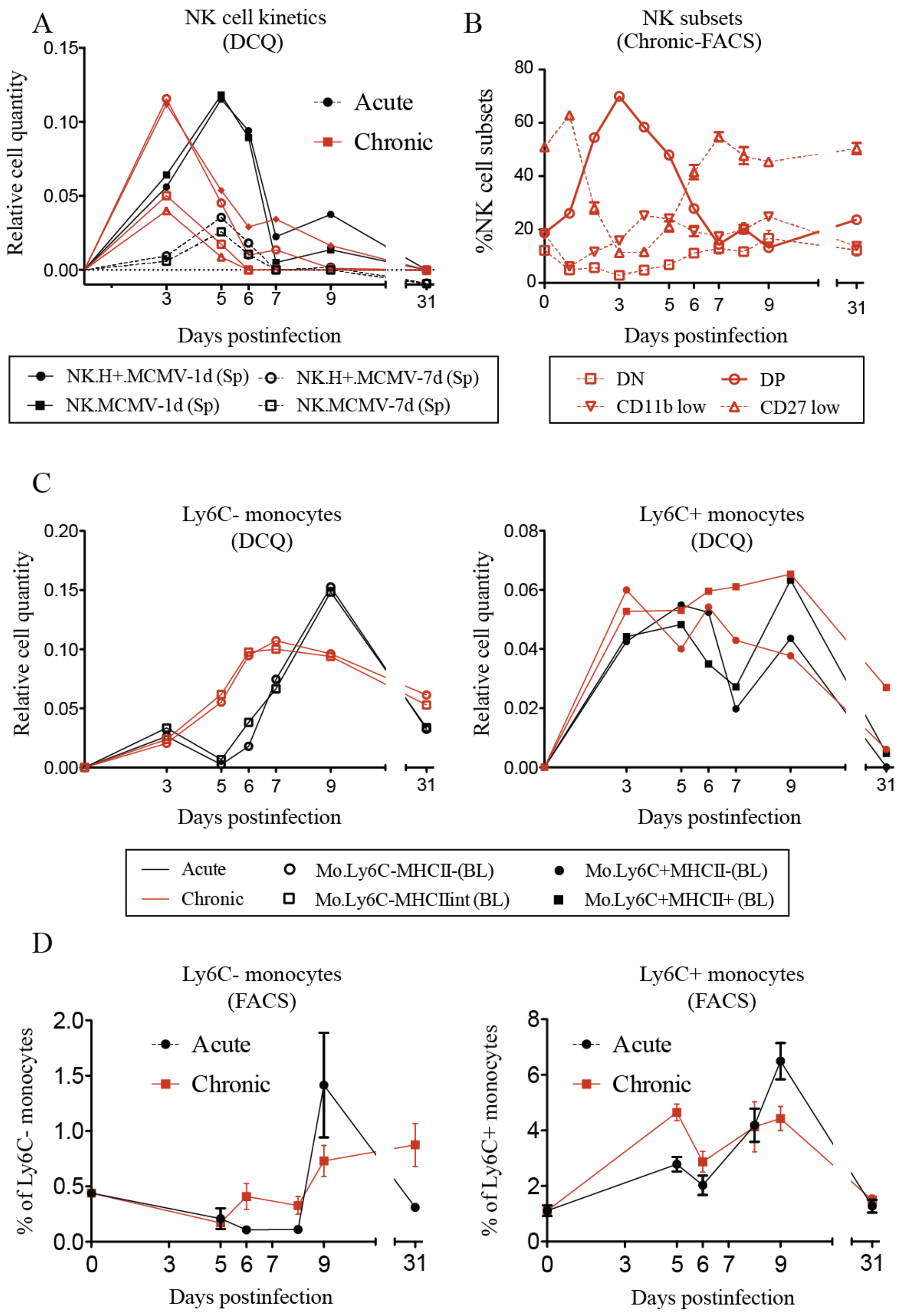

Supplementary Figure 3. Dynamics of NK and monocyte cell subsets. (A) DCQ cell predictions of NK cells from acutely or chronically infected mice. (B) Kinetics of NK cell subsets in chronic infection were analyzed by FACS at the indicated time-points. DN: double negative ($CD11b^{-}CD27^{-}$). DP: double positive ($CD11b^{+}CD27^{+}$). (C) DCQ-predicted cell changes in quantity of monocyte cell subsets. (D) Validation by FACS of cell quantity dynamics of $Ly6C^{-}$ and $Ly6C^{+}$ monocytes in acute and chronic LCMV infection at the indicated time-points.

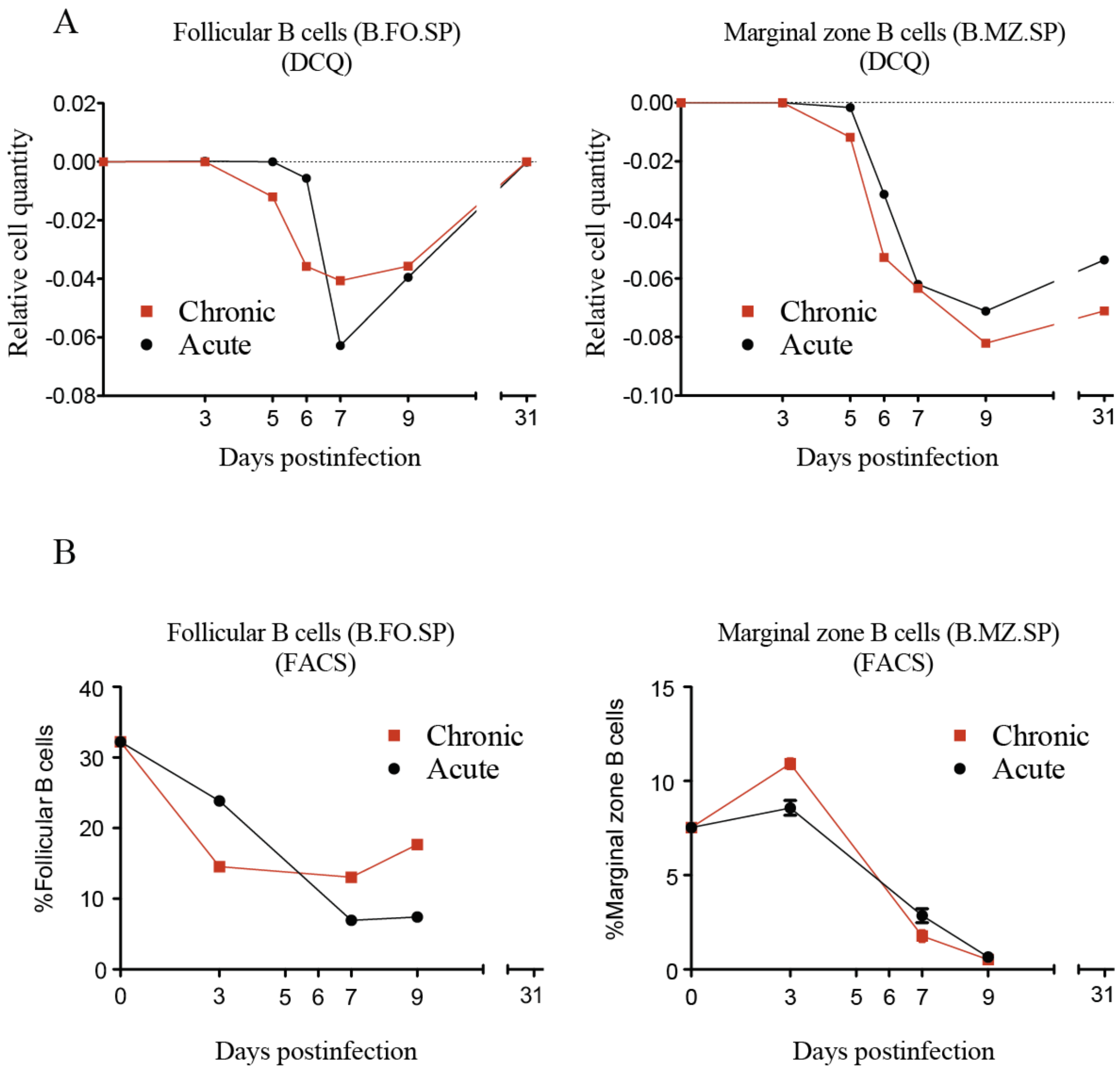

Supplementary Figure 4. Validation of DCQ predicted B cell dynamics. (A) DCQ cell predictions of follicular and marginal zone B cells from acutely or chronically infected mice. (B) Validation by FACS of cell quantity dynamics of follicular and marginal zone B cells in acute and chronic LCMV infection at the indicated time-points.
